# Islamic perspectives in disaster: An alternative to changing fatalistic attitudes

**DOI:** 10.4102/jamba.v12i1.942

**Published:** 2020-11-12

**Authors:** Furqan I. Aksa

**Affiliations:** 1 Department of Geography Education, Samudra University, Aceh, Indonesia

**Keywords:** Islam, disaster, fatalistic, Muslim community, religion

## Abstract

Misunderstandings towards the teachings of Islam increases the fatalistic attitude towards disaster. Fatalistic attitude causes them to ignore measures to reduce the disaster risk. A new approach is needed to change the fatalistic attitude that afflicts most Muslim countries. This article aims to provide an overview of the view of Islam on disasters and the Islamic perspectives on disaster risk reduction. The findings from this literature review reveal that Islamic discourses view disaster as a test from God. There is not a single verse in the Qur’an and the hadith of the Prophet Muhammad who order humans to be fatalistic in their understanding of disasters. On the contrary, Islamic teachings actually give significant attention to the people to use knowledge in disaster risk reduction. This article offers three Islamic principles that can be used in disaster risk reduction, namely *Al-Ilmu* (knowledge), *Ikhtiar* (effort) and *Tawakkul* (trust in God). The concept is expected to fill the limitations of the literature that examines the positive impact of Islamic teachings on disaster risk reduction.

## Introduction

Fatalism is an attitude that believes that everything that happens in the community environment is beyond their control (Ruiu 2012). It is closely related to internal and external controls. According to Rotter (1990), an external monitor occurs when someone assumes that something that happens to him or her is not forever dependent on his or her actions, but rather a fortune or fate. Meanwhile, internal control is a condition when individuals feel that an event depends on their behaviour.

Acevedo (2008) points out that fatalism remains a largely misunderstood phenomenon. It is influenced by political, cultural, religious and historical factors. Acevedo (2008) believes that culture and religion significantly contribute to the formation of the fatalism belief that natural hazards are destiny and their location has been determined by God. In some Muslim-populated countries, religious beliefs often increase fatalistic attitudes towards disasters such as earthquake, volcanic eruption and floods (Baytiyeh & Naja 2014).

In Morocco, most Muslim communities with low education generally tend to link earthquake disasters as divine will (God). They assume that God protects pious people when disasters occur (Paradise 2005). A fatalistic attitude also afflicts high-school students in Turkey and Lebanon. The findings of the research conducted by Baytiyeh and Öcal (2016) reveal that students in Turkey and Lebanon have a high fatalistic attitude towards disasters. The same thing happened in Aceh; massive disaster education carried out after the earthquake and tsunami in 2004 could not change the students’ understanding from the belief that the earthquake and tsunami were a punishment from God (Adiyoso & Kanegae 2012).

A fatalistic attitude results in the lack of disaster preparedness (Baytiyeh & Naja 2016). The higher level of the stronger belief in fatalism in disasters makes the level of preparedness and actions in disaster management lower (Yari, Zarezadeh & Ostadtaghizadeh 2019). Fatalistic beliefs cause humans to ignore measures to reduce the disaster risk.

The high fatalistic attitude in some Islamic countries is thought to be caused by misunderstandings in understanding Islamic teachings (Ghafory-Ashtiany 2014). For example, the public belief that mosques and holy places are resistant to damage has increased the belief in fatalism after the Izmit earthquake in Turkey in 1999 (Ghafory-Ashtiany 2009). Moreover, the misunderstanding of Islamic teachings that have led to an attitude of fatalism is also influenced by the message conveyed by religious leaders. For instance, after the 2004 Indian Ocean earthquake and tsunami, most religious leaders in Aceh interpreted the disaster as a form of punishment from God for human sins (Adiyoso & Kanegae 2013).

Furthermore, the misunderstanding of *Tawakkul* (belief in God) principle also causes fatalism. *Tawakkul* is often interpreted as surrendering to God without the need for effort. This assumption is incorrect because basically God’s will is based on human deeds and behaviour. Ghafory-Ashtiany (2009) points out that without constant struggle, hard work and belief, one cannot hope to achieve perfection and get goodwill from God. The same thing was also pointed out by Fahm (2019) as trusting in God only without preparation is regarded as fatalism.

Therefore, a new approach is needed to change the fatalistic attitude in most Muslim-populated countries. The fatalism of disaster (Act of God, God’s punishment) must be the change. This article would like to provide an overview of the Islamic perspectives on disaster. An understanding of the Islamic perspectives on disasters is expected to be an effective approach to eliminate fatalistic attitudes. An approach that is in accordance with culture and religion is believed to be effective in changing fatalistic attitudes (Ghafory-Ashtiany 2014).

## Disasters in the Islamic perspectives

Referring to the Qur’an, there are three terms that mean disaster: firstly, *musibah*, which means something that befalls human beings in the form of something either pleasant or unpleasant (Zainuddin 2016). In the opinion of most scholars, *musibah* is caused by a human sin. Sin can also be categorised as an evil act that ignores facts and does not use knowledge in an effort to reduce the disaster risk (Chester, Duncan & Dhanhani 2013).

*Secondly*, bala, which means test (human promotion). Bala is God’s will without the human involvement. Bala aims to raise the human standing, forgive his sins and purify his soul (Zainuddin 2016).

[*O*]ne example is the story of Prophet Ibrahim who was tested by God by ordering to slay his beloved son. In essence the command is a test to dignify the Prophet Ibrahim. (Al Quran, ch. 37:102) (Iskandar 2019)

Thirdly, *azab*, which means punishment (punishment of God). In the Qur’an, *azab* is interpreted as torture or a very painful punishment. The punishment is inflicted by God only on those who are ungodly and do not believe. The religious leaders interpret the disaster that befell the people of the previous prophets such as the people of Prophet Shuaib, the people of Noah, the people of Prophet Lut, the People of Prophet Saleh and the tribe ’*Ad* people of Prophet Hud as a punishment for not believing.

From the discussion, it can be concluded that the disasters that occurred in various Islamic countries today can be categorised as a *musibah* from God to test the human faith. This is based on the fact that when a disaster occurs, the victim consists of people who believe and who do not believe in God. One example is that amongst the victims of the disasters such as the 2004 Indian Ocean earthquake and tsunami, there were those who believed and those who did not believe in God. Most of the victims are Acehnese who are Muslims and believe in God. Moreover, the disaster of the 6.5 magnitude earthquake that occurred in Pidie Jaya Aceh in 2016 resulted in a total of 196 mosques collapsed and damaged by the earthquake (BNPB 2019). This can be the proof that disasters can occur at any time and afflict anyone, not just the people who do not believe in God.

## Islamic perspectives in disaster risk reduction

This section will introduce three relevant Islamic concepts used in disaster risk reduction, namely *Al-Ilmu* (knowledge), *Ikhtiar* (effort) and *Tawakkul* (trust in God). *Islam gives great attention to the people to have knowledge. Finding and applying knowledge are a basic requirement for every Muslim* (Abukari 2014). In the view of Islam, humans with significant knowledge have greater advantages. For example, with the knowledge gained, someone can be helpful for others.

In the context of disaster, the hazard-related knowledge can motivate someone to make the right choice when a disaster occurs as carried out by a 10-year-old student from England when the Indian Ocean tsunami struck in 2004. The student, who was on vacation with his family on Phuket Beach, Thailand, managed to save hundreds of lives of people who were vacationing on the beach. The student was able to identify the signs of a tsunami by looking at the seawater that suddenly receded and foam bubbles appeared in the middle of the ocean. This knowledge was obtained from geography lessons at his school two weeks before the tsunami disaster (Aksa et al. 2020; Gregg et al. 2006).

Besides that, refer to the verse Al Quran, chapter 58:11

[*A*]nd when it says: stand ye, then stand up, Allah will exalt the believers among you and those who are given the knowledge of some degree. And Allah knows what you do.

From the explanation of the Qur’an, it can be concluded that Muslims are commanded to gain knowledge for the benefit of the world and thereafter. In the context of disaster risk reduction, the use of science in disaster risk reduction is not prohibited in Islam. Therefore, in the face of disasters, humans need to be equipped with knowledge. The formation of knowledge can be accomplished through the integration of disaster education into the education curriculum. Educational institutions are considered as an environment that can develop a safety culture. It is believed to be able to form better awareness of disaster risk.

*Ikhtiar* means trying seriously to get the best results. In the Islamic view, *Ikhtiar* are things ordered by God, which refers to the verse in Al Quran, chapter 13:11 *Surely Allah will not change the condition of a people, before the people themselves change what is in them.* In the face of disasters, Islam holds that humans are not justified to be fatalistic trying to use all the resources that God has given to reduce the disaster risk. One example is the story of Noah, an inspiring story written in the Qur’an about the importance of trying to prepare you in the event of a disaster.

The story of Noah written in the Qur’an can be a good reference for preparing for disasters (Ghafory-Ashtiany 2009). From the explanation of the verse, it can be learned that God instructed Noah to prepare for the flood that would hit his country. The order can be understood that there is an effort that must be made by humans to reduce the disaster risk.

Also, lexically *Ikhtiar* refers to choice, that is, to choose. This means that humans have the choice to prepare themselves for disasters. For instance, the story of *Umar bin Khattab* – when he was about to enter the outbreak of a disease. His companions asked, *does this action not mean avoiding the destiny set by God? Umar bin Khattab* replied that he avoided a destiny to lead to another destiny (Iskandar 2019). Destiny consists of *qada* and *qadar. Qada* is the destiny that God has written before man was born and cannot be changed. *Qadar* is a destiny that can be changed by trying and praying. Life and death have become God’s provisions so that they cannot be changed. However, humans can still try and pray to avoid disasters that result in death (Ghafory-Ashtiany 2009).

**FIGURE 1:**
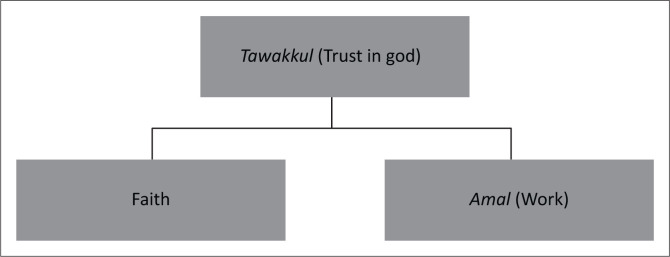
Conceptual framework of *Tawakkul* (trust in God).

However, in the context of a disaster, trust in God without preparedness is considered fatalism (Fahm 2019). Before a disaster occurs, the community must improve preparedness, such as preparing emergency plans, attending workshop-related disaster preparedness, practicing drill disaster preparedness and preparing emergency kits. After all these efforts, people believe in God for the best results. It is related to the conceptual framework of *tawakuul* that consists of components of Faith (trust in God) and *Amal* (work) (Figure 1).

Believing in God means giving to God everything he has tried. In the Islamic view, *Tawakkul* is different from surrender. *Tawakkul* is an active action that requires the maximum effort. The fatalistic attitude that has plagued most Muslim communities in various parts of the world is thought to be wrong in understanding the *Tawakkul* concept. They assume that *Tawakkul* is a form of surrender (surrender to God) without the earnest effort.

The concept of *Tawakkul* originating from the Qur’an is recognised as having a positive impact on the recovery of psychological trauma after a disaster (Dinia et al. 2017). For example, the belief of many Muslims in Aceh about disasters as the destiny of God, there is always convenience after difficulties, the importance of being grateful for what God still gives them, and the belief that the dead are martyrs who will be blessed with a positive impact on healing psychological trauma after the 2004 Indian Ocean earthquake and tsunami disaster. The same thing happened to the people of Yogyakarta, Indonesia. Religious belief (faith) is a source of strength for them to rise after a disaster (Joakim & White 2015).

## Conclusion

The fatalistic attitude that hits most Muslim communities in various countries is the impact of misunderstanding in comprehending Islamic teachings, especially the concept of *Tawakkul* (trust in God). They assume that *Tawakkul* is surrendering to God without having to try and prepare for disaster. This assumption is very wrong. In the Islamic view, *Tawakkul* must begin with hard work from humans. God will not change the fate of humans if humans do not try to change their destiny. Therefore, the use of knowledge and technology in disaster risk reduction is a form of effort that can be performed by humans to reduce the impact of disasters.

The three principles of Islam in disaster risk reduction are expected to be an alternative that can be used to reduce fatalistic attitudes in the Muslim community. In disaster-prone areas, religious beliefs are firmly embedded in the daily lives of their people. Therefore, the concept that is in accordance with the religious beliefs and culture of Islamic society is recognised to be more effectively accepted and utilised for disaster risk reduction.
